# Methane and Carbon Dioxide in the Sediment of a Eutrophic Reservoir: Production Pathways and Diffusion Fluxes at the Sediment–Water Interface

**DOI:** 10.1007/s11270-014-2268-3

**Published:** 2015-02-05

**Authors:** Renata Gruca-Rokosz, Janusz A. Tomaszek

**Affiliations:** Faculty of Civil and Environmental Engineering, Department of Environmental and Chemistry Engineering, Rzeszów University of Technology, al. Powstańców Warszawy 6, 35-959 Rzeszów, Poland

**Keywords:** Methane, Carbon dioxide, δ^13^C-CH_4_, δ^13^C-CO_2_, Sediment

## Abstract

The estimated diffusion fluxes of methane (CH_4_) and carbon dioxide (CO_2_) at the sediment–water interface in the Rzeszów Reservoir in southeastern Poland are presented. The relevant studies were conducted during 2009, 2010, and 2011. Calculated fluxes ranged from 0.01 to 2.19 mmol m^−2^ day^−1^ and from 0.36 to 45.33 mmol m^−2^ day^−1^ for methane and carbon dioxide, respectively. While the values for calculated diffusion fluxes of methane are comparable with those reported for other eutrophic reservoirs, much higher values were obtained here for carbon dioxide. The resulting values of δ^13^C-CH_4_ and the fractionation coefficients between methane and carbon dioxide (αCH_4_-CO_2_) suggest that methane in the sediment of the Rzeszów Reservoir is produced by acetate fermentation, while the hydrogenotrophic methanogenic process is of successively greater importance with increasing depth. In the top layer of the sediment, 24–72 % of CO_2_ came from methanogenesis, while the contribution made by the degradation of organic matter by methanogenesis to CO_2_ was greater in the deeper layer.

## Introduction

Considerable increase of concentrations of greenhouse gases (GHG) in the atmosphere connected with global warming and stratospheric ozone depletion (IPCC 2007), which have been observed in recent years, have led to intensive efforts being undertaken all over the world to quantify GHG emissions from different ecosystems, including aquatic ecosystems (e.g., Xing et al. 2005, Demarty et al. 2009, Delsontro et al. 2010, Gruca-Rokosz et al. (2011a), Bergier et al. 2011). The obtained results of investigations have demonstrated that freshwater ecosystems such as reservoirs are potentially important sources of greenhouse gas emissions. Decomposition of the organic matter accumulated in sediments is an important link in the global carbon cycle, because the products of this process include CO_2_ and CH_4_, both potent gases where the generation and augmentation of the so-called greenhouse effect are concerned. It is estimated that carbon greenhouse gas emissions from reservoirs may account for about 7 % of total emissions from anthropogenic sources (St Louis et al. 2000). This evaluation may be underestimated because it has not been taken into consideration in emissions of gases released to the atmosphere from downstream water of the dams (Guérin et al. 2006).

In conditions of good oxygenation, the final products of the process by which organic matter becomes mineralized are CO_2_ and H_2_O (C_6_H_12_O_6_ + 6O_2_ → 6CO_2_ + 6H_2_O). However, it needs to be recalled that the decomposition process is participated in by oxidants other than oxygen itself, like NO_3_^−^, Fe^3+^, Mn^4+^, and SO_4_^2−^ (Froelich et al. 1979).

Where oxidants are absent, organic matter is also subject to decomposition, by methanogenic bacteria participating in a fermentation process whose final products are CO_2_ and CH_4_ (C_6_H_12_O_6_ → 3CO_2_ + 3CH_4_). Biogenic CH_4_ arises via the two major mechanisms of acetate fermentation (Barker 1936) and CO_2_ reduction (Takai 1970). The former, which is more common in freshwater (sulfate-poor) environments with a large amount of labile organic matter (Piker et al. 1998), involves the hydrolytic decomposition of acetate and generation of CO_2_ and CH_4_ via the reaction CH_3_COOH → CO_2_ + CH_4_. Acetate can also be oxidized to CO_2_ and H_2_O, with the CO_2_ then being reduced metabolically to CH_4_. The source of the electrons is hydrogen: CO_2_ + 4H_2_ → CH_4_ + 2H_2_O (Whiticar 1996). It has been estimated that, in most freshwater ecosystems, acetate fermentation is 50–80 % responsible for the production of methane (Valentine et al. 2004; Bergier et al. 2011).

A reconnaissance of CH_4_ and CO_2_ sources entails research into carbon stable isotopes. Reference to the isotopic composition of dissolved inorganic carbon (DIC) allows the main sources in water to be recognized, be these atmospheric CO_2_, the mineralization of the organic matter present, or the dissolution of carbonates. The latter processes in sediments result in the release to pore water of carbon dioxide isotopically similar to the sources, i.e., to the organic carbon in the sediments and to CaCO_3_. In contrast, CO_2_ released by methanogenesis is enriched in ^13^C as compared with the organic carbon in sediments (Ogrinc et al. 2002).

During hydrogenotrophic methanogenesis, the isotopically lighter carbon species is preferred, with the result that the methane produced via acetate fermentation has δ^13^C-CH_4_ values in the range −65 to −50 ‰, whereas the δ^13^C of methane produced by the reduction of CO_2_ oscillates in the range −110 to −60 ‰ (Whiticar and Faber 1986). The δ^13^C values of the CH_4_ and the CO_2_ coexisting with it are also helpful in determining mechanisms by which methane is generated. The distribution of the carbon isotopes between δ^13^C-CO_2_ and δ^13^C-CH_4_ can be presented as the fractionation factor αCH_4_-CO_2_. The values for αCH_4_-CO_2_ connected with methanogenesis in a marine environment—where the main pathway of methane formation is the reduction of CO_2_—are in the range 1.05–1.1. In contrast, in the freshwater ecosystems where acetate fermentation predominates, the values for this indicator range between 1.04 and 1.05 (Whiticar 1996).

While available literature yields a fair amount of information on CO_2_ and CH_4_ emissions to the atmosphere from the surfaces of reservoirs in different climatic zones, there is a paucity of information on fluxes of these gases at the sediment–overlying water interface. The small amounts of data probably reflect the methodological difficulties arising in regard to the collection, extraction, and measurement of concentrations of these gases in the pore water of sediments. Fluxes of CH_4_ and CO_2_ to the atmosphere via the water–air interface are not usually equivalent to fluxes of these gases from sediments, because proportions of the two gases emitted to the atmosphere from the water–air interface can be modified markedly by microbiological processes. Exhaustive information on the production of greenhouse carbon gases in bottom sediments, and on their transport to the overlying water, is therefore crucial to the overall carbon balance, representing a valuable enhancement of knowledge on the role of the small, eutrophic reservoirs occurring so commonly around the world where the emission of greenhouse gases is concerned, and hence also possible global warming.

The goal of the work was to determine values for the diffusive fluxes of CO_2_ and CH_4_ at the sediment–water interface, as well as the pathways of these gases in bottom sediments, using the results of research into their concentrations and carbon isotopic compositions in the pore and overlying waters of a small, severely degraded reservoir.

## Materials and Methods

### Study Area

The Rzeszów Reservoir in southeastern Poland was constructed in 1974 by damming of the Wisłok River in 63 + 760 km of its course. The reservoir is supplied by two main tributaries: Wisłok and Strug. Its main purpose was to allow for the proper operation of the water supply for the city of Rzeszów. Because of its the location on the outskirts of a large city, it fulfills a vital role as a sports and recreation lagoon. The total volume of the reservoir decreased by 0.7 mln m^3^ of its capacity during last 40 years. Consequently, the reservoir has mostly silted up and gradually transformed into land especially in its upper zone. Double attempts to rehabilitate the usability of the reservoir have not brought the expected results.

The Rzeszów reservoir watershed covers an area of 2,050 km^2^. The Wisłok flows through the foothill areas that are largely agricultural, though the upper parts are forested, while the middle part is lined with industrial centers (glassworks, tanneries, refineries). The catchment of a smaller tributary, the Strug, is vastly agricultural in nature which traditionally is comprised of fragmented farmland representing high population density. The reservoir is under strong anthropopressure associated with local agriculture that caused a severe erosion of the land, as a result of depositing the rubble and local source contaminations (Koszelnik and Tomaszek 2002).

**Fig. 1:**
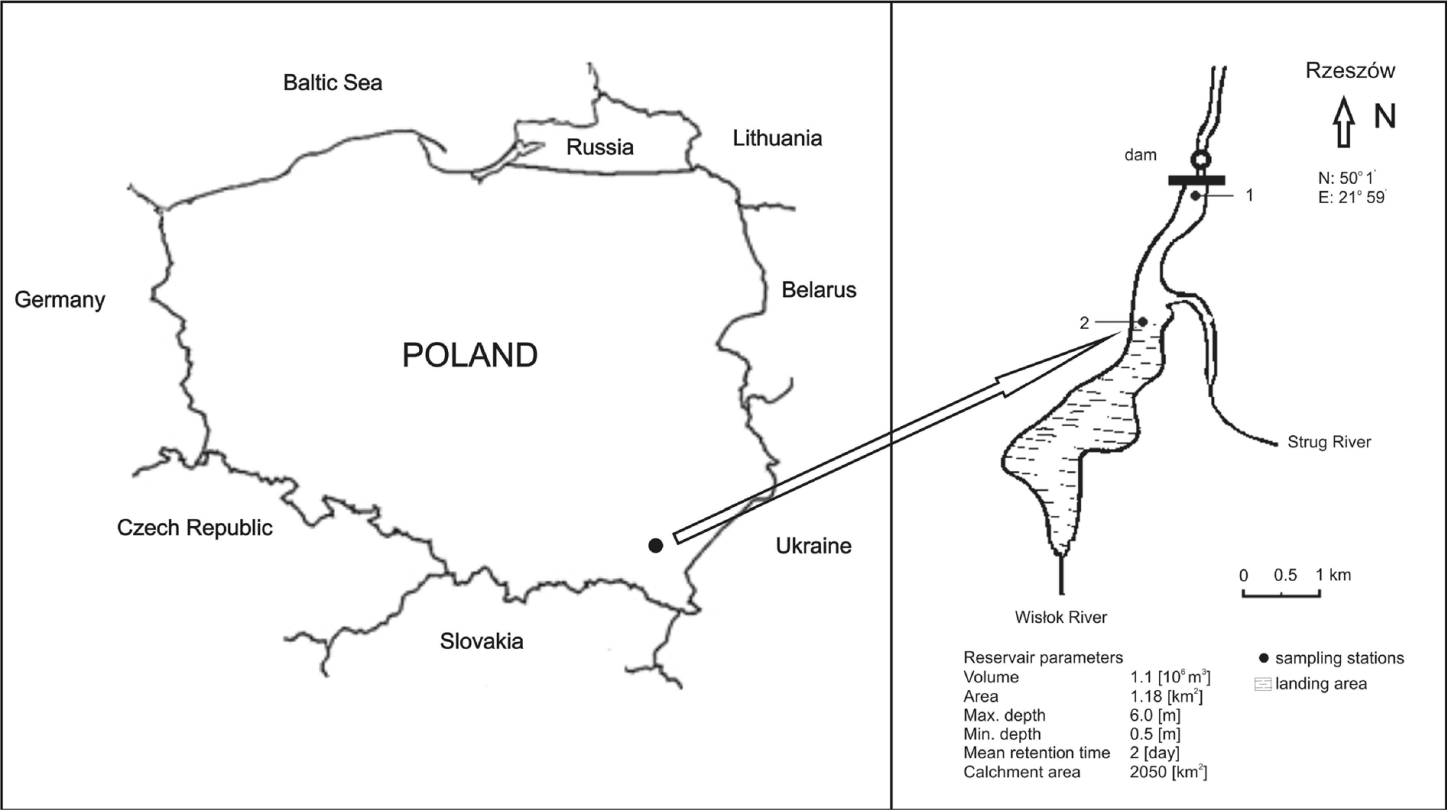
Localization of the Rzeszów Reservoir with sampling stations

Two-point characteristics of the reservoir as a whole were chosen for study. Station 1 was located near the dam, whereas station 2 was in the zone of the main tributary, immediately beyond the point of entry into the reservoir. The research station areas were lacking in vegetation. The locations of the sampling stations are as shown in Fig. 1.

### Sediment and Water Sampling and Preparation

The studies were carried out during 2009, 2010, and 2011. Samples of sediment and surface water were sampled 11 times between May and November (in 2009, once in October; in 2010, five times in May, June, July, September, and November; in 2011, five times in May, June, July, August, and November). Sediment cores were being taken from the littoral using a gravity (KC Kajak of Denmark) sediment corer. Surface water samples were collected to 0.5-dm^3^ plastic bottles. Sampled surface water and cores together with overlying water were subject to immediate transport to the laboratory. Although sediment cores are normally processed for gases in helium-filled glove bags, the non-measurement of nitrogen ensured that cores were processed in the open, within a few hours of collection. Sedimentary cores were progressively pushed out from the bottom of Plexiglas tubes by a piston, and top (1 cm) layers of the sediment were placed in a modified pore water squeezer (Reeburgh 1967). Three times during the study (in May, July, and September 2011), pore water samples from deeper layers of sediment (1–3, 3–5, 5–10, and 10–15 cm) were also extruded. The pore water obtained was collected directly in gastight glass vials, in order for contact with the atmosphere to be avoided. The surface and overlying water were also collected into gastight glass vials, using a polypropylene syringe connected to a hose. Immediately after collection, the samples of water in the vials were acidified using 6 N HCl (final concentration ∼50 mM) to quantitatively convert all carbonate anions into CO_2_ (Miyajima et al. 1995).

### Surface Water Analysis

Temperature was measured in situ with a MultiLine P4 (WTW, Germany). Total phosphorus (TP) and total nitrogen (TN) were determined spectrophotometrically (photoLAB S12, WTW, Germany) in non-filtrated and mineralized samples of water (TN—the salicylate method, coefficient of variation of the procedure (CVP) ±1.5 %; TP—reaction with ammonium molybdate, CVP ±1.6 %). Chlorophyll “a” was determined spectrophotometrically (photoLAB S12, WTW, Germany) after hot extraction to ethanol (CVP ±2.5 %).

### Pore Water and Overlying Water Analysis

Gas concentrations and stable carbon isotopic compositions in the pore and overlying water were analyzed using a headspace equilibration technique. Gases were extracted from the water into gastight glass vials, through the displacement of a known volume of water using helium. Water was equilibrated in the vials with added helium by means of 5 min of vigorous shaking. Then, gas samples were taken from headspace and analyzed for concentrations of CH_4_ and CO_2_ and δ^13^C-CH_4_ and δ^13^C-CO_2_.

Concentrations of both CH_4_ and CO_2_ were measured using a Pye Unicam gas chromatograph with analytical error of ±5 % (model PU-4410/19) equipped with a flame ionization detector (FID) and a stainless steel column packed with a Haye Sep Q, 80/100 Mesh, 6 ft in length and of 2 mm ID. The GC was also equipped with a methanizer to detect low levels of carbon dioxide. The methanizer is packed with a nickel catalyst powder and heated to 380 °C. When the column effluent mixes with the FID hydrogen supply and passes through the methanizer, CO_2_ is converted to CH_4_. The carrier gas was helium at a flow rate of 30 cc/min. Gas concentrations were expressed in micromoles per decimeter of gas in the water.

The carbon isotopic compositions of CH_4_ and CO_2_ were determined using gas chromatograph combustion isotope mass spectrometry (GC-CIII-IRMS DELTA^Plus^ Finnigan). The isotope ratios were expressed in δ-notation (δ^13^C): δ^13^C = (^13^C / ^12^C_(sample)_ / ^13^C / ^12^C_(standard)_ − 1] · 10^3^, relative to the Pee Dee Belemnite (PBB) standard. The precision of measurement was about ±0.3 ‰ for δ^13^C-DIC and ±0.5 ‰ for δ^13^C-CH_4_.

### Sediment Analysis

For porosity measurements, the water content per volume of sediment was determined by drying a known volume of the wet sediment to a constant weight at 105 °C. The pH of sediment in the suspension with 1 N KCl was determined potentiometrically with a MultiLine P5m (WTW, Germany) (Ostrowska et al. 1991). The organic matter (OM) was analyzed by the loss on ignition (LOI) method at 550 °C for 4 h.

Before the analysis of organic carbon (OC), total nitrogen (TN), δ^13^C, and δ^15^N, carbonates were removed from the samples by 72 h contact with the vapor of 30 % HCl in desiccators (Zimmermann et al. 1997). The OC and TN concentrations were subsequently measured using an analyzer of carbon and nitrogen (CN Flash EA 1112, ThermoQuest) at 1,020 °C. Blank and standard samples with known elemental composition (sulfanilamide) were used for quality control. The precision of the method was about ±3 %. Stable isotopic compositions of the organic carbon and total nitrogen were determined using an IRMS DELTA^Plus^ Finnigan on line with the analyzer of carbon and nitrogen (CN Flash EA 1112, ThermoQuest). The isotopic ratios were reported in standard δ-notation (δ^13^C, δ^15^N) expressed as “per mil”: δ*R* (‰) = (*R*_a_/*R*_b(sample)_ / *R*_a_/*R*_b(standard)_ − 1] · 10^3^, where *R*_a_/*R*_b_ are the ^13^C/^12^C or ^15^N/^14^N ratios relative to the PDB and AIR standards, respectively. The methods were calibrated using International Atomic Energy Agency (IAEA-NO3) standard for δ^15^N and National Bureau of Standards 22 (NBS 22) for δ^13^C. The precision of measurements was ±0.1 ‰ for δ^13^C and ±0.4 ‰ for δ^15^N.

### Calculations

The diffuse fluxes of pore water gases from sediments were calculated using Fick’s first law of diffusion:

1 $$ J=-\phi {D}_{\mathrm{s}}\left(\mathrm{dc}/\mathrm{d}\mathrm{z}\right) $$

where *J* is the diffusive flux, *ϕ* is the porosity, *D*_s_ is the sediment diffusion coefficient for each individual gas, and dc/dz is the concentration change for each gas with depth.

*D*_s_ was calculated in two ways: according to Berner (1980) and according to Lerman (1979). The arithmetic average of two calculations was used for the diffusion values. The difference between the arithmetic average and the values obtained from each way of calculation was ±15 %.

According to Berner:

2 $$ {D}_{\mathrm{s}}={D}_0{\theta}^{-2} $$

where *D*_0_ is the molecular diffusion coefficient in pure water and *θ* ^2^ the tortuosity of sediments (with sediment tortuosity estimated using the empirical relationship developed by Sweerts (1990) for freshwater environments:

3 $$ {\theta}^2=\left.-0.73\phi +2.17\right) $$

According to Lerman:

4 $$ {D}_{\mathrm{s}}={D}_0{\phi}^2 $$

where *D*_0_ is the molecular diffusion coefficient in pure water, and *ϕ* is sediment porosity.

*D*_0_ diffusion coefficients for CH_4_ in water were calculated using linear interpolation between values 0.95 × 10^−5^ cm^2^ s^−1^ (5 °C) and 1.5 × 10^−5^ cm^2^ s^−1^ (20 °C) (Lerman 1979). *D*_0_ values for CO_2_ in water were calculated after Hobler (1986). The concentration gradient was determined between the value in the water just above the sediment–water interface and the first pore water gas measurement (approximately. 1-cm-depth interval).

Isotopic fractionation factor for conversion of CO_2_ to CH_4_ is defined as:

5 $$ {\upalpha \mathrm{C}\mathrm{H}}_4-{\mathrm{CO}}_2=\left({\updelta}^{13}\mathrm{C}-{\mathrm{CO}}_2 + 1,000\right)/\left({\updelta}^{13}\mathrm{C}-{\mathrm{CH}}_4+1,000\right) $$

where δ^13^C-CO_2_ and δ^13^C-CH_4_ are the isotopic composition of CO_2_ and CH_4_, respectively.

Relative contribution of hydrogenotrophically derived CH_4_ to total CH_4_ was determined by mass balance equation (Conrad et al. 2010a):

6 $$ {\mathrm{fCH}}_{4,\mathrm{h}}=\left({\updelta}^{13}\mathrm{C}-{\mathrm{CH}}_4-{\updelta}^{13}\mathrm{C}-{\mathrm{CH}}_{4,\mathrm{a}}\right)/\left({\updelta}^{13}\mathrm{C}-{\mathrm{CH}}_{4,\mathrm{h}}-{\updelta}^{13}\mathrm{C}-{\mathrm{CH}}_{4,\mathrm{a}}\right) $$

where fCH_4,h_ is being the fraction of CH_4_ formed by hydrogenotrophy, δ^13^C-CH_4_ is the δ^13^C of total produced methane, and δ^13^C-CH_4,a_ and δ^13^C-CH_4,h_ are the δ^13^C of methane derived from acetoclastic and hydrogenotrophy methanogenesis, respectively. The δ^13^C-CH_4,a_ and δ^13^C-CH_4,h_ values were calculated using αCH_4_-CO_2_ obtained by Whiticar (1996) and δ^13^C-CO_2_. In this calculation, two different αCH_4_-CO_2_ values were used, with values of 1.04 and 1.07 for acetotrophy and hydrogenotrophy, respectively.

The calculations of sharing of CO_2_ originating from methanogenesis were based on the isotopic mass balance. It was assumed that the process of fermentation of the organic matter deposited in bottom sediments would entail the generation of approximately similar amounts of CH_4_ and CO_2_: CH_3_COOH → CO_2_ + CH_4_ (Barker 1936), thus:

7 $$ {\updelta}^{13}\mathrm{C}-\mathrm{O}\mathrm{M}=0.5{\updelta}^{13}\mathrm{C}-{\mathrm{CH}}_4+0.5{\updelta}^{13}\mathrm{C}-{\mathrm{CO}}_{2\left(\mathrm{methanogenesis}\right)} $$

where δ^13^C-OM is δ^13^C of the organic matter, δ^13^C-CH_4_ is δ^13^C of the methane, and δ^13^C-CO_2(methanogenesis)_ is δ^13^C of the carbon dioxide derived from methanogenesis.

By transforming the formula, it was possible to calculate the value of δ^13^C-CO_2_ formed by methanogenesis.

The fraction of CO_2_ originating in the process of methanogenesis was determined using the mass balance equation (Corbett et al. 2013):

8 $$ f=\left({\updelta}^{13}\mathrm{C}-{\mathrm{CO}}_{2\left(\mathrm{pore}\ \mathrm{water}\right)}-{\updelta}^{13}\mathrm{C}-{\mathrm{CO}}_{2\left(\mathrm{O}\mathrm{M}\kern0.5em \mathrm{decay}\right)}\right)/\left({\updelta}^{13}\mathrm{C}-{\mathrm{CO}}_{2\left(\mathrm{methanogenesis}\right)}-{\updelta}^{13}\mathrm{C}-{\mathrm{CO}}_{2\left(\mathrm{O}\mathrm{M}\kern0.5em \mathrm{decay}\right)}\right) $$

where *f* is the participation of CO_2_ derived from methanogenesis, δ^13^C-CO_2(pore water)_ δ^13^C of CO_2_ measured in pore water, and δ^13^C-CO_2(OM decay)_ the value of δ^13^C for carbon dioxide originating through the mineralization of organic matter. In the calculations, it was assumed that δ^13^C-CO_2(OM decay)_ is equal to δ^13^C-OM, because mineralization of organic matter releases inorganic carbon into the pore water, this being isotopically similar to the source, i.e., to sedimentation organic carbon (Ogrinc et al. 2002).

### Statistical Analysis

For the obtained results, basic descriptive statistics such as the minimum, maximum, average, and standard deviation values were calculated. The MS Excel 2007 program was used for calculations. For linear relationships, Pearson’s correlation coefficient with the corresponding level of significance *p* was calculated. A Student’s *t* test was used to compare means for the two groups (the sampling stations). It was performed using the Statistica 10 PL Statistical Package. Significances were defined as *p* < 0.05.

## Results

### Sediment Characteristics

**Fig. 2:**
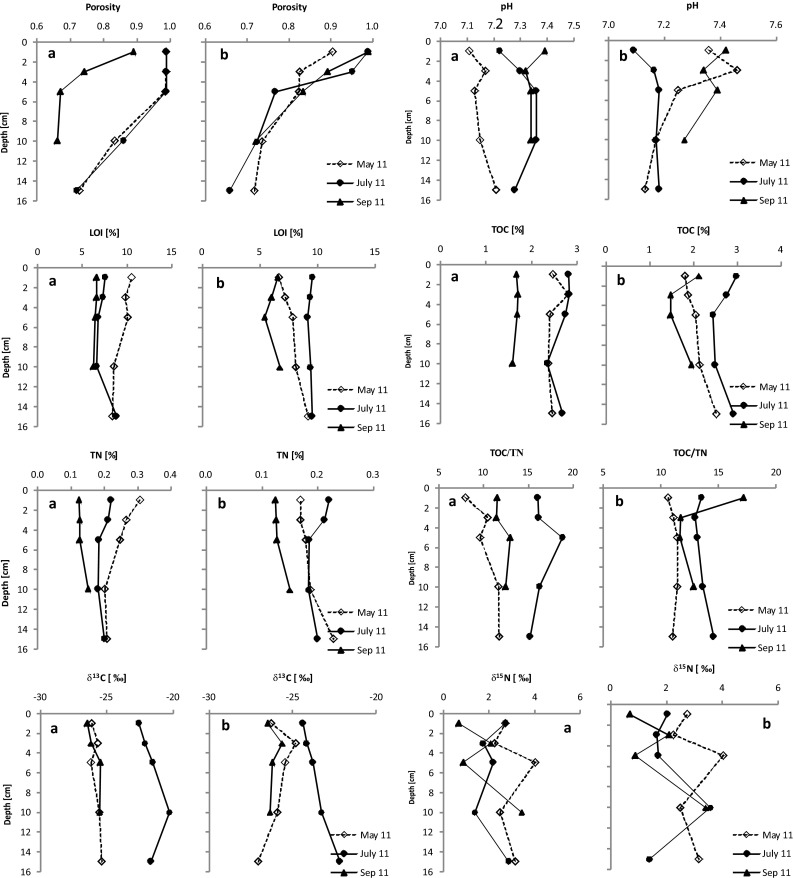
Vertical profile of selected parameters in sediment of the Rzeszów Reservoir (**a** station 1, **b** station 2)

The surface layer of the sediments studied has high porosity in the range 0.84 to 0.99 at station 1 and 0.79 to 0.99 at station 2 (Fig. 2). Obtained values did not attest to statistically significant differences between stations (*t* = 0.4437; *p* = 0.6622). At progressively greater depths, the porosity of the sediments decreases—to reach a value of 0.66 at both stations some 10–15 cm down into the sediment layer (Fig. 2). The reaction of the sediments only slightly exceeded a value of 7, with no statistically significant differences between stations (*t* = −0.2640; *p* = 0.7946). A minimal pH value of 7.04 was noted in June 2011 at station 1, in the surface layers of the sediments. This compared with a maximum value of 7.46 observed in May 2011 at station 2 in the 1–3-cm layer of sediment (Fig. 2). The organic matter content in the surface layer was not high, in the range 6.54–11.58 % (Fig. 2), and these values did not manifest statistically significant differences between stations (*t* = −0.2303; *p* = 0.8203). Furthermore, changes in the content of organic matter with depth did not achieve significance either (Fig. 2). The content of total organic carbon (TOC) deposited in the surface layers of sediments was similar at the two research stations (*t* = −0.4138; *p* = 0.6836) and constituted around 2.3 % of the dry mass of the sediments on average (Fig. 2). As with organic matter, no significant differences in levels of TOC down profiles were observed (Fig. 2). However, TOC in the studied sediments was closely correlated with the content of organic matter (*R* ^2^ = 0.89, *p* < 0.001), with the former on average accounting for some 25 % of the latter. The contribution of total nitrogen in both the surface layers of sediments and down the profiles was quite well aligned and varied between 0.12 and 0.37 % (Fig. 2). Statistically significant differences between the stations were not noted (*t* = 0.0021; *p* = 0.9983). The mean contents of total nitrogen at the two stations were similar, at 0.21 %. Furthermore, in the case of both the δ^15^N and δ^13^C values measured in sediments, no statistically significant differences between stations were to be observed (*t =* −1.3209; *p* = 0.2022—for δ^15^N; *t =* 1.0303; *p* = 0.3158—for δ^13^C). δ^15^N values in the surface layer of sediments fell within the range −0.1 to 3.2 ‰, while δ^13^C values were between −29.2 and −22.6 ‰ (Fig. 2).

From analysis of *δ* values in sediment profiles, it is clear that there is almost always depletion as regards the ^12^C isotope at increased depth. The mean increase for δ^13^C was thus about 1 ‰ between the surface of sediments and a depth of 15 cm. An exception was provided by the situation noted in May 2011 at station 2, in that the δ^13^C value was lower 15 cm down than at the surface by about 0.8 ‰. However, to a depth of 10 cm down sediments, higher values of δ^13^C were also observed. Values for δ^15^N in the analyzed profiles followed different patterns, there being a wide variety of values between 0.5 and 4.2 ‰, with no unambiguously defined trends to be noted (Fig. 2).

### Characteristics of the Analyzed Waters

**Fig. 3:**
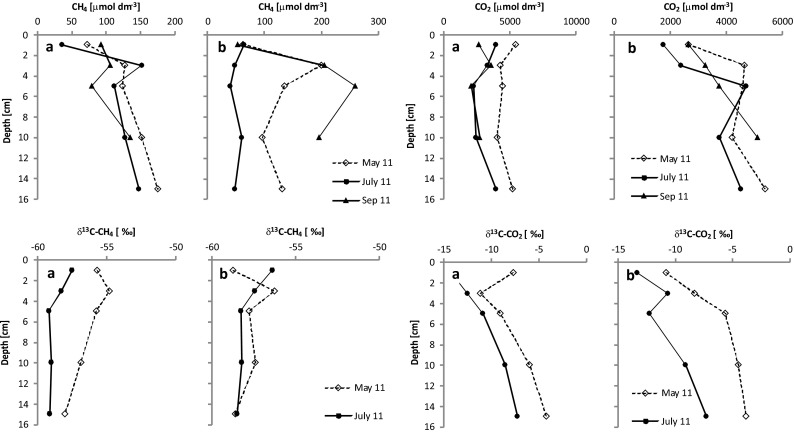
Vertical profiles for methane and carbon dioxide concentrations and δ^13^C-CH_4_ and δ^13^C-CO_2_ values in pore water of the Rzeszów Reservoir (**a** station 1, **b** station 2)

Concentrations of methane in the pore water (of the 0–1-cm sediment layer) varied within the range 7.25–232 μmol dm^−3^ (Table 1 and Fig. 3). The mean values for concentrations at the two research stations were similar, oscillating around 75 μmol dm^−3^. A lack of statistically significant differences was confirmed by the Student’s *t* test (*t* = −0.0955; *p* = 0.9249). With one exception, at both research stations, the CH_4_ concentration in pore water increased with depth (Fig. 3). Carbon dioxide concentration in the examined pore water was much higher than that of methane, varying in the range 1,118–5,466 μmol dm^−3^ (Table 1 and Fig. 3). No statistically significant differences were noted between the stations (*t* = 1.0605; *p* = 0.3022). At station 1, small differences in CO_2_ concentrations at different depths were observed, whereas at station 2, there was more than a doubling of concentrations between depths of 1 and 10–15 cm (Fig. 3). During the whole period of research, average concentrations of CH_4_ and CO_2_ were lower in overlying water than in pore water, falling within the ranges 0.55–36 and 1,063–2,781 μmol dm^−3^, respectively (Table 1). And while mean values for concentrations of the two gases were higher at station 1, statistical analysis did not reveal significant differences between the stations (*t =* −1.5116; *p* = 0.1471 for CH_4_ and *t =* 0.2239; *p* = 0.8329 for CO_2_).

**Table 1:** Methane and carbon dioxide concentrations and δ^13^C-CH_4_ and δ^13^C-CO_2_ values in pore water (0–1-cm depth) and overlying water and water temperature, total phosphorus, total nitrogen, and chlorophyll “a” concentrations in surface water of the Rzeszów Reservoir

	Station 1				Station 2			
	Min	Max	Average	SD	Min	Max	Average	SD
CH_4_ [μmol dm^−3^] (*n* = 10–St 1) (*n* = 11–St 2)	7.25^a^ 0.55^b^	232.00^a^ 36.00^b^	74.51^a^ 8.12^b^	64.47^a^ 10.19^b^	20.00^a^ 5.45^b^	158.67^a^ 25.09^b^	76.73^a^ 13.49^b^	40.02^a^ 5.69^b^
CO_2_ [μmol dm^−3^] (*n* = 10–St 1) (*n* = 11–St 2)	1,118^a^ 1,063^b^	5,466^a^ 2,781^b^	3,106^a^ 1,800^b^	1,393^a^ 495^b^	1,181^a^ 1,116^b^	3,733^a^ 2,509^b^	2,585^a^ 1,758^b^	808^a^ 404^b^
δ^13^C-CH_4_ [‰]	−61.2^a^ (*n* = 7) –50.8^b^ (*n* = 1)	−55.7^a^ (*n* = 7) –50.8^b^ (*n* = 1)	−58.9^a^ (*n* = 7)	2.0^a^ (*n* = 7)	−60.2^a^ (*n* = 9) –56.8^b^ (*n* = 5)	−53.6^a^ (*n* = 9) –48.7^b^ (*n* = 5)	−56.8^a^ (*n* = 9) –52.6^b^ (*n* = 5)	1.9^a^ (*n* = 9) 3.6^b^ (*n* = 5)
δ^13^C-CO_2_ [‰]	−18.6^a^ (*n* = 8) –18.4^b^ (*n* = 8)	−7.6^a^ (*n* = 8) –9.7^b^ (*n* = 8)	−13.2^a^ (*n* = 8) –12.9^b^ (*n* = 8)	3.1^a^ (*n* = 8) 2.6^b^ (*n* = 8)	−13.3^a^ (*n* = 9) –19.4^a^ (*n* = 9)	−7.7^a^ (*n* = 9) –8.8^b^ (*n* = 9)	−10.7^a^ (*n* = 9) –12.7^b^ (*n* = 9)	1.8^a^ (*n* = 9) 3.3^b^ (*n* = 9)
t [°C] (*n* = 12)	8	24.3	17.7	4.7	7.0	24.2	17	4.8
P_tot_[mg P dm^−3^] (*n* = 12)	0.09	1.53	0.28	0.40	0.09	1.49	0.27	0.39
N_tot_[mg N dm^−3^] (*n* = 12)	1.26	3.56	2.26	0.68	1.26	3.39	2.04	0.74
Chl "a"[μg dm^−3^] (*n* = 12)	0.00	112.54	28.47	38.99	1.48	22.21	8.33	6.63

^a^pore water (0–1-cm depth)

^b^overlying water

δ^13^C-CH_4_ values measured in the pore water of the surface layer of sediments were in the ranges −61.2 to −53.6 ‰ (Table 1), and statistically significant differences between the stations were observed (*t* = −2.3784; *p* = 0.0311).

Analysis of changes in δ^13^C-CH_4_ values with depth of sediments shows greater depth to be associated with depleted of carbon as regards the ^13^C isotope. The decrease in δ^13^C (in all but one case) was 2 ‰ on average between the surface of the sediments and a depth of 15 cm (Fig. 3). δ^13^C-CH_4_ values measured in the overlying water varied in the range −56.8 to −48.7 ‰ (Table 1) and were higher than those measured in the pore water—undoubtedly in connection with the oxidation of methane diffusing into the overlying water from the zones of generation.

In the case of δ^13^C-CO_2_ measured in the pore water of the 0–1-cm layer of sediment, statistically significant differences between research stations were again lacking (*t =* −2.0896; *p* = 0.0541). With depth (from the surface of sediments to 15 cm), the δ^13^C value increased 6 ‰ on average (Fig. 3).

δ^13^C-CO_2_ values in overlying water ranged from −19.4 to −8.8 ‰ (Table 1). The mean values at both research stations were almost identical and amounted to about −13 ‰. The standard deviations were 3.1 and 1.8 ‰ for station 1 and station 2, respectively. The lack of statistically significant differences between the stations was confirmed by the Student’s *t* test (*t* = −0.1034; *p* = 0.9190).

Concentrations of total phosphorus measured in surface water were similar at the two research stations (*t* = 0.0873; *p* = 0.9312) (Table 1). Incidental high values (of about 1.5 mg dm^−3^) were observed at both sampling stations in June 2010, this reflecting abundant rainfall and a flood which took place in later months. Apart from these cases, total phosphorus concentrations at the two stations ranged from 0.09 to 0.3 mg dm^−3^. According to Vollenveider (1968), the threshold total phosphorus concentration beyond which the mass development of algae can take place is 0.015 mg dm^−3^—making it clear that algal blooms were potentially possible in the reservoir during the entire period of study.

Concentrations of total nitrogen likewise presented no statistically significant differences between stations (*t* = 0.7575; *p* = 0.4567). The lowest value noted was 1.26 mg dm^−3^, while the highest did not exceed 3.6 mg dm^−3^ (Table 1).

The concentration of chlorophyll “a” in surface water ranged from 0 to above 112 μg dm^−3^ (Table 1). Obtained values did not differ significantly between stations (*t* = 1.6875; *p* = 0.1063), though the mean concentration of chlorophyll was higher at the station located in the dam part of the reservoir.

Analysis of concentrations of total forms of biogenic elements and chlorophyll “a” in line with the criteria for assessing trophic status from the OECD and Nürnberg (Vollenweider and Kerekes 1982; Nürnberg 2001) pointed to a very unfavorable trophic situation for the waters of the Rzeszów Reservoir. Annual average concentrations of total phosphorous and total nitrogen are such as to classify water at the two stations as hypertrophic. The annual average and maximal values for the concentration of chlorophyll “a” also point to the water at the station near the dam being hypertrophic, while values for the upper part of the reservoir indicate a eutrophic or even mesotrophic state. The calculated phosphorus (TSI TP) and chlorophyll (Chla TSI) trophic indices after Carlson (1977), respectively, indicated a hypertrophic or eutrophic status of the water. This must be considered to reflect the supply of huge amounts of nutrients via the main tributaries: the Wisłok and Strug. Earlier estimates showed that the mean loading of the Rzeszów Reservoir with biogenic compounds is about 3,500 mg N m^−2^ day^−1^ and 285 mg P m^−2^ day^−1^ (Koszelnik and Tomaszek 2002), this considerably exceeding the “dangerously high” values proposed by Vollenweider and Kerekes (1982) (1.36 mg N m^−2^ day^−1^ and 0.09 mg P m^−2^ day^−1^, respectively).

## Discussion

### Diffusive Fluxes of CH_4_ and CO_2_ at the Sediment–Water Interface

**Fig. 4:**
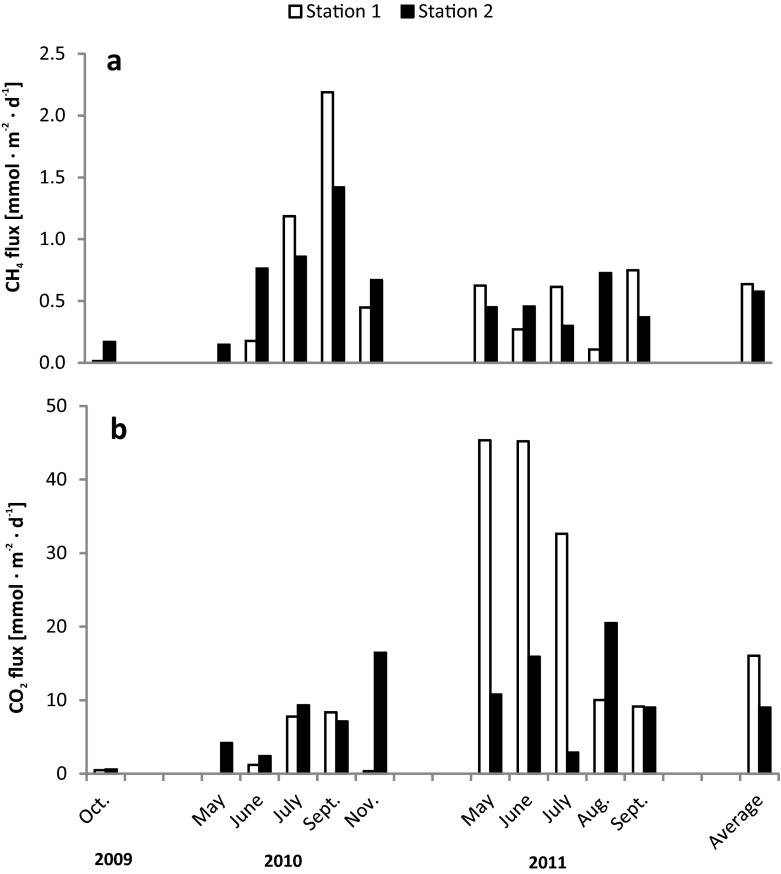
Diffusive fluxes of CH_4_ (**a**) and CO_2_ (**b**) at the sediment–water interfaces of the Rzeszów Reservoir. 11 May 2010, station 1—not measured

Values for calculated diffusive fluxes of methane and carbon dioxide at the sediment–overlying water interface are as shown in Fig. 4. The methane diffusion flux was low, falling within the range 0.01–2.19 mmol m^−2^ day^−1^. The mean values for the fluxes at the two research stations were similar, amounting to 0.64 and 0.58 mmol m^−2^ day^−1^, respectively. The lack of differences in the methane flux between stations was confirmed by statistical analysis (*t* = 0.2757; *p* = 0.78570).

Values for the diffusive flux of CO_2_ at the sediment–water interface were higher, ranging from 0.36 to 45.33 mmol m^−2^ day^−1^. The average value for the flux at station 1 was 16.05 mmol m^−2^ day^−1^, at station 2 9 mmol m^−2^ day^−1^. Despite the visible differences in mean values for fluxes, statistical analysis did not reveal significant differences between the stations (*t* = 1.2194; *p* = 0.2376).

There was no observed dependence between the obtained values for the fluxes and either season of the year or water temperature. The calculated fluxes for methane and carbon dioxide were compared with values obtained by other researchers, making it clear that those for CH_4_ resembled findings from other eutrophicated reservoirs (falling within the range 0.2–19.27 mmol m^−2^ day^−1^, according to Adams (2005). The same author gives lower values for CO_2_ fluxes than CH_4_ fluxes in eutrophic reservoirs, the ranging being −0.06 to 17.70. In our case, the recorded values were much higher. Ogrinc et al. (2002) and Casper et al. (2003) also obtained higher flows for CO_2_ than CH_4_ (Table 2). To make this comparison clearer and more complete, other values for diffusive fluxes of methane and carbon dioxide reported in the literature for the sediment–overlying water interface in different water environments are compiled together in Table 2.

**Table 2:** Diffusive fluxes of CH_4_ and CO_2_ in different aquatic environments (ranges or averages)

	CH_4_ fluxes (mmol m^−2^ day^−1^)	CO_2_ fluxes (mmol m^−2^ day^−1^)	References
Solina Reservoir (Poland)		1.08–1.51	Gruca-Rokosz et al. (2011b)
Wilcza Wola Reservoir (Poland)	0.01–0.14	1.14–2.27	Gruca-Rokosz et al. (2011b)
Tuusulanjärvi Lake (Finland)	4.50		Huttunen et al. (2006)
Postilampi Lake (Finland)	6.56		Huttunen et al. (2006)
Soiviojärvi Lake (Finland)	0.54		Huttunen et al. (2006)
Takajärvi Lake (Finland)	0.30		Huttunen et al. (2006)
Luminakajärvi Lake (Finland)	1.69		Huttunen et al. (2006)
Ranuajärvi Lake (Finland)	4.75		Huttunen et al. (2006)
Lokka Reservoir (Finland)	0.03		Huttunen et al. (2006)
Porttipahta Reservoir (Finland)	1.56		Huttunen et al. (2006)
Bled Lake (Slovenia)	2.20	5.10	Ogrinc et al. (2002)
Orta Lake (Italy)	0.13–7.37		Adams and Baudo (2001)
Stechlin Lake (Germany)	0.05–0.20	2.30–3.40	Casper et al. (2003)

### Sources of CH_4_ and CO_2_ in Bottom Sediments

δ^13^C-CH_4_ values in the bottom sediments of the Rzeszów Reservoir ranged from ca. −61 to ca. −53 ‰ (Fig. 3 and Table 1), with a lowering of the *δ* value to be observed at greater depth (Fig. 3). At the same time, the δ^13^C-CO_2_ value ranged from about −19 to −4 ‰ (Fig. 3 and Table 1), higher values being observed at greater depths in sediments (Fig. 3). The measured values for δ^13^C-CO_2_ were the result of mixing of CO_2_ deriving from oxygen-induced mineralization of organic matter, the dissolution of carbonates, and methanogenesis. Carbonates are characterized by high values for δ^13^C (Conrad et al. 2009; Ogrinc et al. 2002), so their dissolution could be assumed to make a significant contribution to the formation of CO_2_ at greater depths in the sediments. In such a situation, the value of δ^13^C-CO_2_ should theoretically increase with lower pH, though such a relationship was not to be noted in our case. In connection with the above information and with the fact that CO_2_ generated by methanogenesis is enriched in ^13^C in relation to the organic carbon in sediments (Ogrinc et al. 2002), it was hypothesized that the higher quantity of CO_2_ at greater depths in sediments results from the process of methane production.

The value for δ^13^C-CH_4_ is commonly used to indicate the sources of methane in bottom sediments. As noted above, methane is mainly generated through acetate fermentation or else CO_2_ reduction. In the bottom sediments of the Rzeszów Reservoir, both superficially and in the deeper layers, the highest noted value for δ^13^C-CH_4_ was of about −53 ‰, while the lowest was ca. −61 ‰. Such values obtained for δ^13^C-CH_4_ are characteristic for freshwater reservoirs (Nüsslein et al. 2003; Lima 2005), and they attest to methane in the dam reservoir under study being generated thanks to acetate fermentation.

**Fig. 5:**
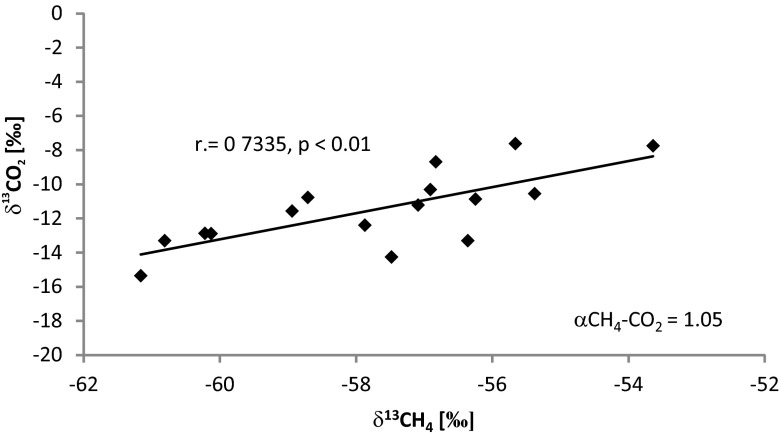
δ^13^C-CH_4_ vs. δ^13^C-CO_2_ in pore water (0–1 cm) of the Rzeszów Reservoir (stations 1 and 2)

In recognizing the mechanisms underpinning CH_4_ production, it is also helpful to know the values of δ^13^C and the CO_2_ coexisting alongside it (Fig. 5). In the top (0–1 cm) layer of the bottom sediments of the studied reservoir, the value of the coefficient of fractionation αCH_4_-CO_2_ over the whole research period was 1.05, this confirming earlier considerations arising on the basis of δ^13^C-CH_4_ values and indicating that methane is produced as a result of acetate fermentation.

Calculations using Eq. 6 of methane produced as a result of CO_2_ reduction demonstrated that acetate fermentation predominated (63–81 %) at the surface layer (0–1 cm) of sediments during the entire period of investigations (Table 3).

**Table 3:** The calculated contribution of CH_4_ hydrogenotrophic in total CH_4_

	CH_4_ hydrogenotrophic [%]					
	Date/depth	0–1 cm	1–3 cm	3–5 cm	5–10 cm	10–15 cm
Station 1	20 October 2009	35				
	16 June 2010	35				
	14 July 2010	36				
	16 September 2010	30				
	17 May 2011	37	21	32	47	58
	15 June 2011	28				
	12 July 2011	20	29	38	46	51
Station 2	20 October 2009	29				
	11 May 2010	35				
	16 June 2010	38				
	14 July 2010	32				
	16 September 2010	28				
	17 May 2011	37	37	52	55	61
	15 June 2011	25				
	12 July 2011	19	33	30	41	49
	09 August 2011	29				

In the deeper sediment layers, an increase in the importance of hydrogenotrophic formation of the CH_4_ was observed. In the 10–15-cm sediment layer, CO_2_ reduction slightly predominated over the acetate fermentation in spring, while in the summer, the contribution of both mechanisms in the methane formation was comparable and approximated 50 %. According to Mandic-Mulec et al. (2012), in the surface sediment layer, predominance of CO_2_/H_2_ pathway was slightly more than 50 % and at a depth of 15 cm was almost 90 %.

No statistically significant correlation was found between the mechanisms of the CH4 creation and water temperature. It is probable that temperature change does not exert a significant influence on the mechanisms by which methane is formed. The influence of temperature on the mechanisms of the CH4 formation is not ambiguous. Nüsslein et al. (2003) have drawn similar conclusions to ours, whereas Lojen et al. (1999) argue that the mechanisms underpinning methane formation in freshwater ecosystems depend on the season of year, with acetate fermentation dominating in spring (65 %) and the reduction of CO_2_ in autumn (about 95 %). Mandic-Mulec et al. (2012) also demonstrated that hydrogenotrophic formation of the CH_4_ prevailed in December with temperature of 6 °C. Dominance of the hydrogenotrophic pathway was observed both in the ice-covered lake of East Antarctica (Wand et al. 2006) and the lakes in the Amazon, where the water temperature over the sediments was around 30 °C (Conrad et al. 2010b). In the light of the above, temperature is not the dominant factor influencing on methanogenic pathways in freshwater environment.

According to Hornibrooke et al. (2000), the mechanism of methane production in deep sediments lacking fresh, more labile organic matter moves in the direction of CO_2_ reduction. These conclusions are in line with those from our research. Values for αCH_4_-CO_2_ were lower in deeper layers of the sediments and below 10 cm reached the value of 1.06 characteristic for hydrogenotrophic methanogenic processes. The calculated contribution of CH_4_ hydrogenotrophic in total CH_4_ also gained importance with increasing sediment depth. To confirm these conclusions, the origin of organic matter in sediment cores was analyzed, by reference to TOC/TN ratios, the assumption being that values in excess of 12 relate to matter of terrestrial origin, while those below 8 concern autochthonous matter (Martinotti et al. 1997; Gąsiorowski and Sienkiewicz 2013). In the event, values for the TOC/TN ratio in the sediment cores studied ranged from 8 to 19 (Fig. 2). Only in July did the obtained results tend to indicate a terrigenous origin of organic matter (at station 1 especially). The other results point to the organic matter deposited being of mixed origin. The participation of autochthonous matter in the sediments studied was determined on the basis of the two sources model (Murase and Sakamoto 2000). Calculations made use of TOC/TN values obtained, while reference values obtained from the literature are 6.8 and 17.1, respectively, in the cases of planktonic and terrigenous matter (Koszelnik 2009). The calculations showed that in most cases, the share of autochthonous organic matter grew smaller with depth, ambiguous results being obtained only in July at station 1. For example, in May at station 1, autochthonous organic matter in the surface (0–1 cm) layer of sediments took an 88 % share, as compared with 52 % in the deeper (10–15 cm) layer.

The kind of organic matter undoubtedly influences the processes by which methane is formed. Prior research has shown that aquatic ecosystems characterized by high levels of primary production offer more favorable conditions for the generation of methane (Furlanetto et al. 2012). Algae decompose to methane and carbon dioxide ten times faster than lignocelluloses (Benner et al. 1984), this making it clear that autochthonous organic matter is a better substratum for methanogenesis (Gruca-Rokosz et al. 2011a).

Also attesting to the fact that the kind of organic matter deposited in bottom sediments can play an important role as regards, not only the amounts of methane produced but also the mechanism by which the gas is generated is research carried out by Murase and Sugimto (2001) The values these authors obtained for both δ^13^C-CH_4_ and αCH_4_-CO_2_ in the bottom sediments of the lake they studied were characteristic for marine environments rather than freshwater and clearly showed that methane was being created through the reduction of CO_2_. It should be emphasized that the lake they studied was oligo/mesotrophic rather than eutrophic (as has been the case for most of the ecosystems described) and was also poor in autochthonous organic matter. The seasonal influence of organic matter quality on the mechanism of methane formation was confirmed by other researchers. According to Lojen et al. (1999), the sediments investigated during the spring contained considerable amount of planktonic—easily biodegradable organic matter which fostered acetate fermentation. Mandic-Mulec et al. (2012) have found that an increase in the significance of hydrogenotrophy with the depth of sediments was linked to the absence of labile organic matter.

To confirm the earlier hypothesis about the increased role of methanogenesis in CO_2_ production in the deeper layers of sediments, the share of CO_2_ originating from this process was determined (Eq. 8). The remaining results of the calculations are as shown in Table 4.

**Table 4:** The calculated contribution of CO_2_ made by the degradation of organic matter via methanogenesis

	Methanogenesis [%]					
	Date/depth	0–1 cm	1–3 cm	3–5 cm	5–10 cm	10–15 cm
Station 1	20 October 2009	58				
	16 June 2010	47				
	14 July 2010	42				
	16 September 2010	24				
	17 May 2011	63	50	58	63	65
	15 June 2011	31				
	12 July 2011	24	27	29	30	39
Station 2	20 October 2009	65				
	11 May 2010	49				
	16 June 2010	72				
	14 July 2010	55				
	16 September 2010	52				
	17 May 2011	48	53	61	68	74
	15 June 2011	47				
	12 July 2011	35	41	33	41	41
	09 August 2011	72				

The surface 1-cm layer of bottom sediments thus has between 24 and as much as 72 % of its carbon dioxide deriving from methanogenesis, with values not found to depend on either temperature or season of the year. The contribution of methanogenesis to the process of carbon dioxide generation is found to be greater deeper down in the layer of sediment. Similar research results have been obtained by other researchers. In research by Kelly et al. (1982), CO_2_ produced during methanogenesis was found to account for 70–80 % of the total, in Lojen et al. (1999), the value was about 43 %, and in Ogrinc et al. (2002), it ranged between 38 and 78 %, clearly prevailing in the deeper layers of sediment and in the anaerobic parts of the lake. In Corbett et al. (2013), as with our results, the contribution of CO_2_ generated by methanogenesis was greater further down in the layers of sediment, reaching 36 % at a depth of 10 cm, 61 % at a depth of 50 cm, 56 % at the surface of the sediments, and 75 % at a depth of 64 cm. In the surface layer of sediment, Ogrinc et al. (2002) only observed a predominance of CO_2_ originating from methanogenesis in summer, when the temperature of sediments was higher and there was more of the labile organic matter derived from microalgae and phytoplankton. In the deeper, anaerobic parts of the sediments, the season of year did not appear to be of any significance.

## Conclusion

The diffusion fluxes calculated for CH_4_ and CO_2_ at the bottom sediment–overlying water interfaces fall in the ranges from 0.01 to 2.19 and 0.36–45.33 mmol m^−2^ day^−1^, respectively. In the case of CH_4_, they reach values characteristic for other eutrophic reservoirs, while the values noted for the fluxes of CO_2_ are significantly greater than those invoked in describing eutrophic bodies of water. No dependent relationship between values for diffusion fluxes and temperature or season of the year was to be observed.

Carbon isotopes were used to determine the origin of the examined gases in bottom sediments. The obtained values for δ^13^C-CH_4_ and αCH_4_-CO_2_ in pore water suggest that these sediments were mainly generating methane through fermentation, albeit with CO_2_ reduction assuming greater importance in the production of the gas at greater depths in the sediment. The results suggest that the mechanism underpinning methane formation is influenced by the type of organic matter. Favorable to acetate fermentation is the presence of the fresh, more labile organic matter usually deposited in the surface layer of bottom sediments.

The research carried out showed that between 24 and 72 % of the CO_2_ in the top layer of the studied sediments was produced by methanogenesis. No relationship was found between the contribution of methanogenesis to CO_2_ formation and the season of the year and temperature. However, the results do suggest that the role of methanogenesis in CO_2_ production increases further down into the reservoir sediments.
